# Supplementary material for: WRN promotes bone development and growth by unwinding SHOX-G-quadruplexes via its helicase activity in Werner Syndrome
Source: Nat Commun. 2022 Sep 16;13:5456. doi: 10.1038/s41467-022-33012-6 (PMC9481537; doi:10.1038/s41467-022-33012-6)
Supplement: Supplementary file 1 — Supplementary Information [file 41467_2022_33012_MOESM1_ESM.pdf]

WRN promotes bone development and growth by unwinding SHOX-G-quadruplexes via its helicase activity in Werner Syndrome

Yuyao Tian[1], Wuming Wang[1,2], Sofie Lautrup[3], Hui Zhao[1,4], Xiang Li[5], Patrick Wai Nok Law[1], DINH Ngoc Duy[6], Evandro Fei Fang[4], Hoi Hung Cheung[1], Wai-Yee Chan[1,2,4*]

[1] School of Biomedical Sciences, Faculty of Medicine, the Chinese University of Hong Kong, Shatin, N.T., Hong Kong SAR.
[2] CUHK-SDU University Joint Laboratory on Reproductive Genetics, the Chinese University of Hong Kong, Shatin, N.T., Hong Kong SAR.
[3]Department of Clinical Molecular Biology, University of Oslo and Akershus University Hospital, 1478 Lørenskog, Norway
[4] Hong Kong Branch CAS Center of Excellence for Animal Evolution and Genetics, the Chinese University of Hong Kong, Shatin, N.T., Hong Kong SAR.
[5] CAS Key Laboratory of Tissue Microenvironment and Tumor, Shanghai Institute of Nutrition and Health, University of Chinese Academy of Sciences, Chinese Academy of Sciences
[6]Department of Biomedical Engineering, the Chinese University of Hong Kong, Shatin, N.T., Hong Kong SAR.

Contents

1. Supplementary figures
2. Supplementary tables

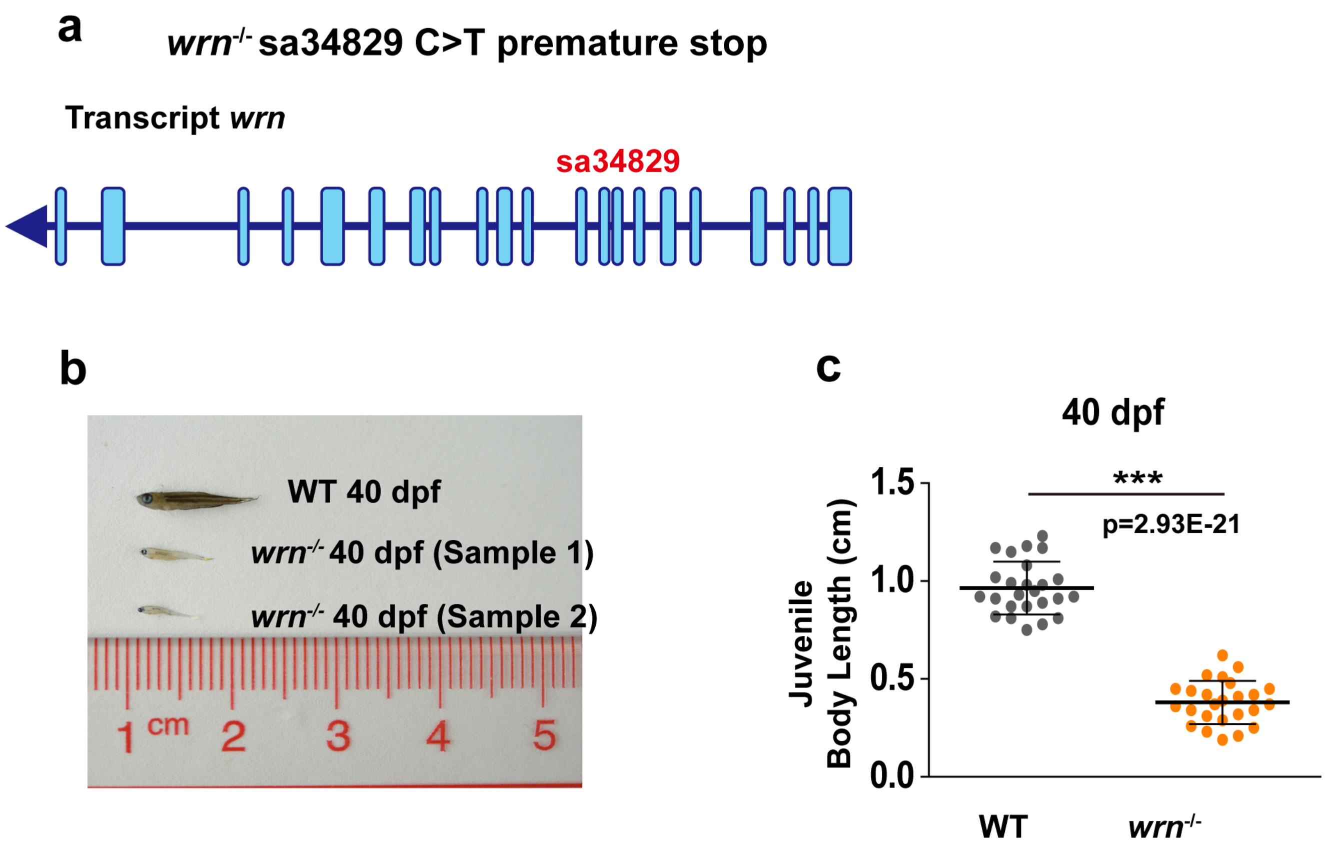
a
wrn-/- sa34829 C>T premature stop
Transcript wrn
sa34829
b
WT 40 dpf
wrn-/- 40 dpf (Sample 1)
wrn-/- 40 dpf (Sample 2)
1 cm 2 3 4 5
c
40 dpf
***
p=2.93E-21
Juvenile Body Length (cm)
1.5
1.0
0.5
0.0
WT
wrn-/-

Supplementary Fig. 1. Generation of *wrn*$^{-/-}$ mutant zebrafish. a. Diagram of ENU method of point mutation (*wrn*^{sa34829}, C>T, premature stop) generating *wrn*$^{-/-}$ mutant zebrafish. Information was based on ZFIN websites (https://zfin.org/ZDB-ALT-160601-3425). b. Representative bright-field images of WT and *wrn*$^{-/-}$ mutant zebrafish of three independent experiments at 40 dpf. c. Dot graph analysis of the total body length at 40 dpf. N=25 independent juvenile zebrafish for WT and *wrn*$^{-/-}$ mutants respectively. Each dot represents a biological replicate. Statistical analysis was performed using two-tailed unpaired Student's t-test. $*P < 0.05$, $**P < 0.01$, $***P < 0.001$. 3 independent biological experiments were performed.

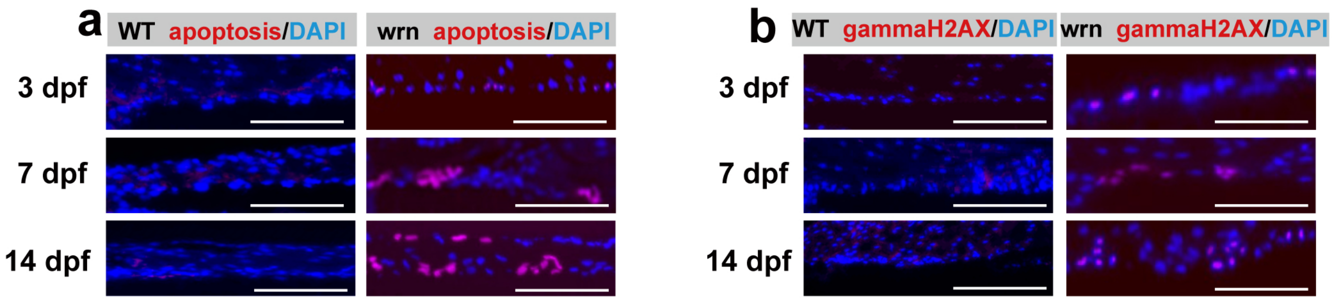
a
WT apoptosis/DAPI
wrn apoptosis/DAPI
3 dpf
7 dpf
14 dpf
b
WT gammaH2AX/DAPI
wrn gammaH2AX/DAPI
3 dpf
7 dpf
14 dpf

Supplementary Fig. 2. Loss of wrn causes DNA damage and apoptosis *in vivo*. a. Representative *In situ* apoptosis images of three independent experiments in WT and *wrn*$^{-/-}$ mutant zebrafish at 3 dpf, 7 dpf, and 14 dpf. Scale bar = 100 μm. b. Representative images of three independent experiments of γH2AX expression in WT and *wrn*$^{-/-}$ mutant zebrafish at 3 dpf, 7 dpf, and 14 dpf. Scale bar = 100 μm.

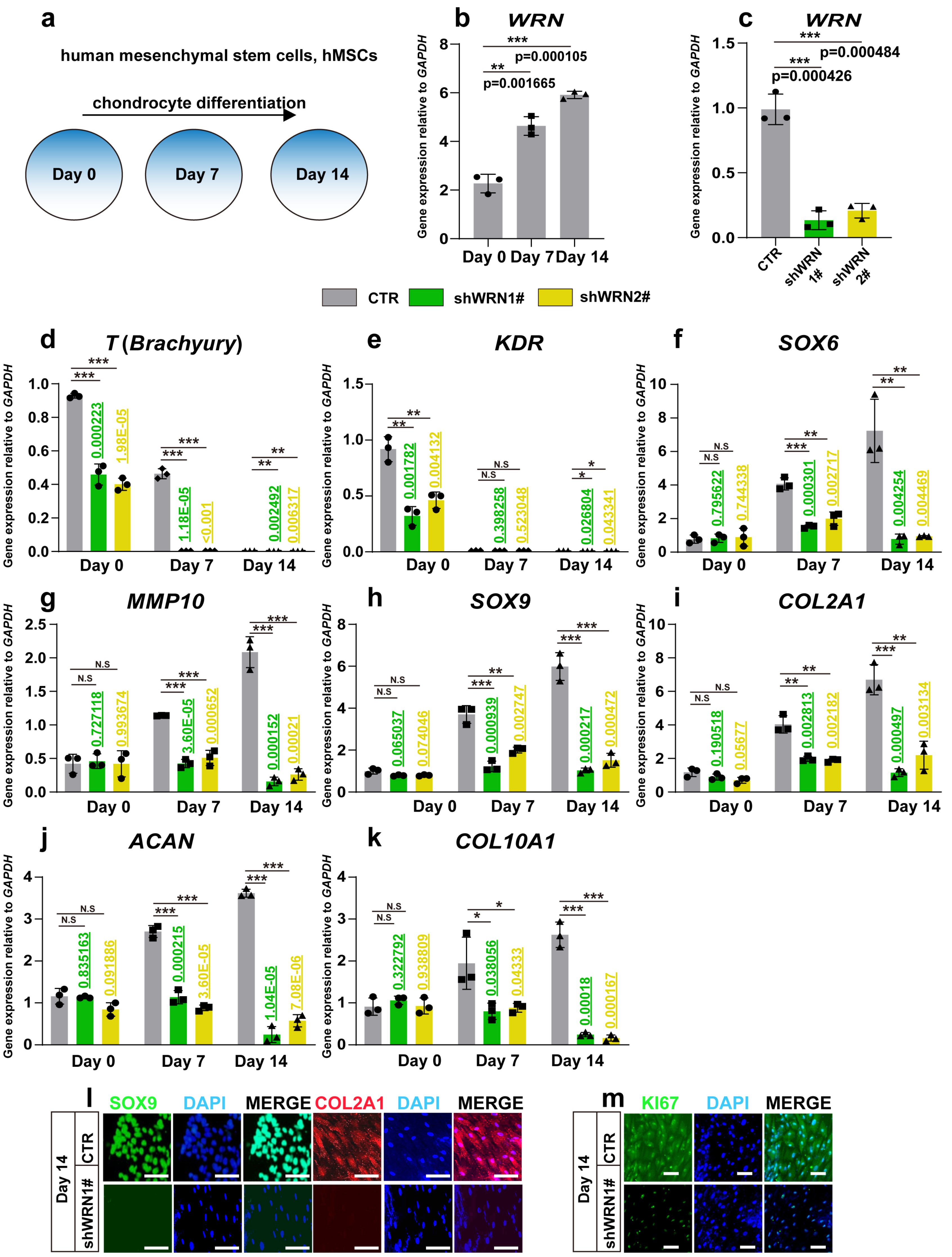
a
human mesenchymal stem cells, hMSCs
chondrocyte differentiation
Day 0
Day 7
Day 14
b
WRN
p=0.000105
p=0.001665
Gene expression relative to GAPDH
Day 0
Day 7
Day 14
c
WRN
p=0.000484
p=0.000426
CTR
shWRN 1#
shWRN 2#
CTR
shWRN1#
shWRN2#
d
T (Brachyury)
0.000223
1.98E-05
1.18E-05
<0.001
0.002492
0.006317
e
KDR
0.001782
0.004132
0.398258
0.523048
0.026804
0.043341
f
SOX6
0.795622
0.744338
0.000301
0.002717
0.004254
0.004469
g
MMP10
0.727118
0.993674
3.60E-05
0.000652
0.000152
0.00021
h
SOX9
0.065037
0.074046
0.000939
0.002747
0.000217
0.000472
i
COL2A1
0.190518
0.05677
0.002813
0.002182
0.000497
0.003134
j
ACAN
0.835163
0.091886
0.000215
3.60E-05
1.04E-05
7.08E-06
k
COL10A1
0.322792
0.938809
0.038056
0.04333
0.00018
0.000167
N.S
l
SOX9
DAPI
MERGE
COL2A1
DAPI
MERGE
Day 14
CTR
shWRN1#
m
KI67
DAPI
MERGE
Day 14
CTR
shWRN1#

Supplementary Fig. 3 Depletion of WRN inhibits chondrogenesis in hMSCs. a. The illustration of human mesenchymal stem cells (hMSCs) model during chondrogenesis. b. qRT-PCR measurement of *WRN* expression profile during chondrogenesis in hMSCs with N = 3 independent biological experiments. c. qRT-PCR measurement of *WRN* knockdown efficiency in hMSC model. N = 3 independent biological experiments. d-k. qRT-PCR measurement of genes related to mesodermal stages (*T* and *KDR*) and chondrocytes (*SOX9*, *COL2A1*, *SOX6*, and *COL10A1*) in hMSCs. N = 3 independent biological experiments. l. Representative immunofluorescent staining of three independent experiments in the CTR and shWRN1# groups on day 14 in hMSCs. SOX9 and COL2A1 were examined. Scale bar = 50 μm. m. Representative immunofluorescent staining of three independent experiments in the CTR and shWRN1# groups on day 14 in hMSCs. Ki67 was examined. Scale bar = 20 μm. Data are presented as the mean ± S.D. Statistical analysis was performed using two-tailed unpaired Student's t-test. *$P < 0.05$, **$P < 0.01$, ***$P < 0.001$.

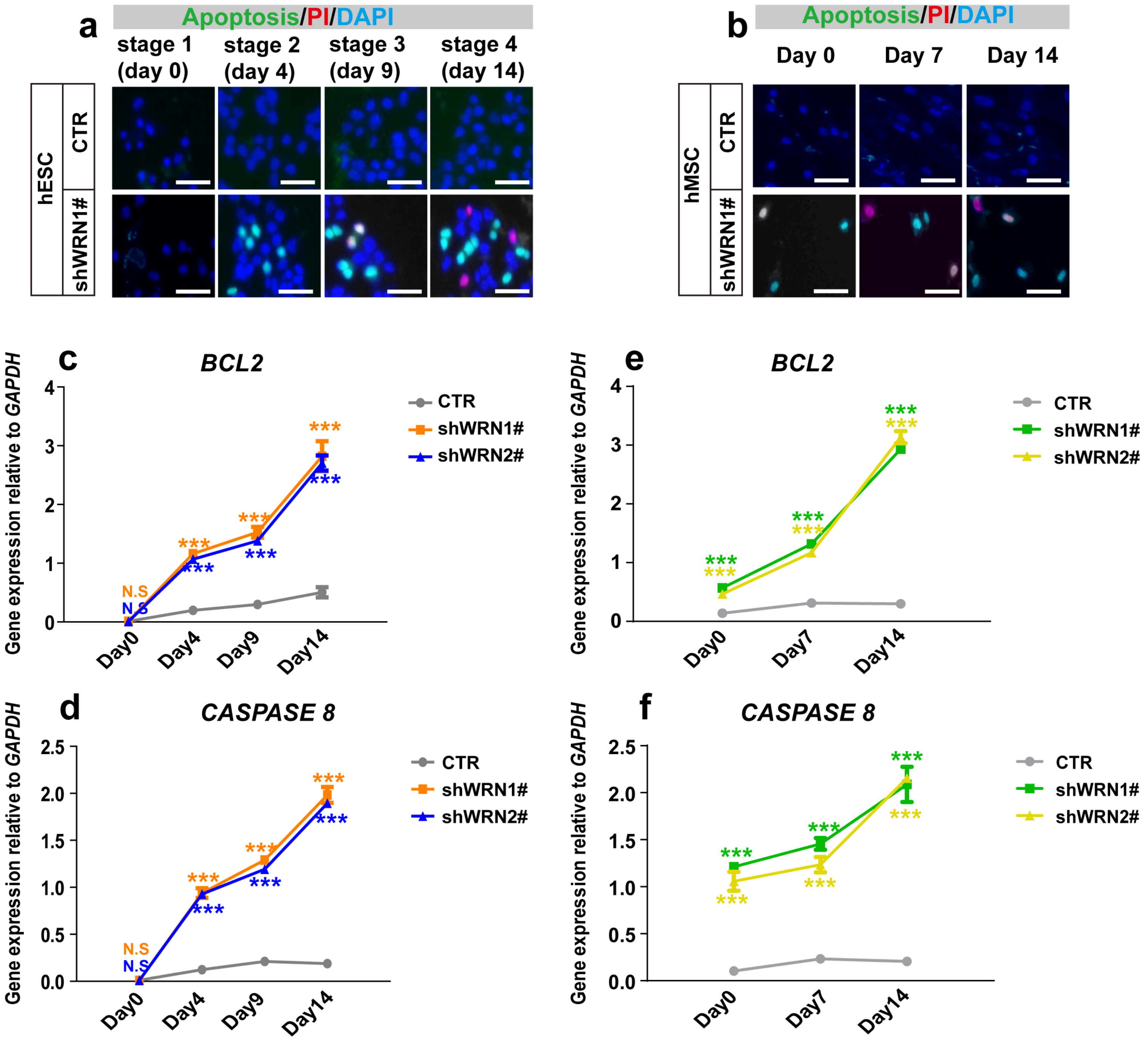

a
Apoptosis/PI/DAPI
stage 1 (day 0)
stage 2 (day 4)
stage 3 (day 9)
stage 4 (day 14)
hESC
CTR
shWRN1#
b
Apoptosis/PI/DAPI
Day 0
Day 7
Day 14
hMSC
CTR
shWRN1#
c
BCL2
Gene expression relative to GAPDH
CTR
shWRN1#
shWRN2#
N.S
Day0
Day4
Day9
Day14
d
CASPASE 8
Gene expression relative to GAPDH
CTR
shWRN1#
shWRN2#
N.S
Day0
Day4
Day9
Day14
e
BCL2
Gene expression relative to GAPDH
CTR
shWRN1#
shWRN2#
Day0
Day7
Day14
f
CASPASE 8
Gene expression relative to GAPDH
CTR
shWRN1#
shWRN2#
Day0
Day7
Day14

Supplementary Fig. 4. Loss of *WRN* leads to apoptosis *in vitro*. a-b. Representative apoptosis and PI staining of three independent biological experiments in hESC and hMSC. Scale bar = 50 μm. c-f. qRT-PCR measurement of *BCL2* and *CASPASE 8* both in hESC and hMSC on day 14. N = 3 independent biological experiments. Data are presented as the mean ± S.D. Statistical analysis was performed using two-tailed unpaired Student's t-test. **P* < 0.05, ***P* < 0.01, ****P* < 0.001.

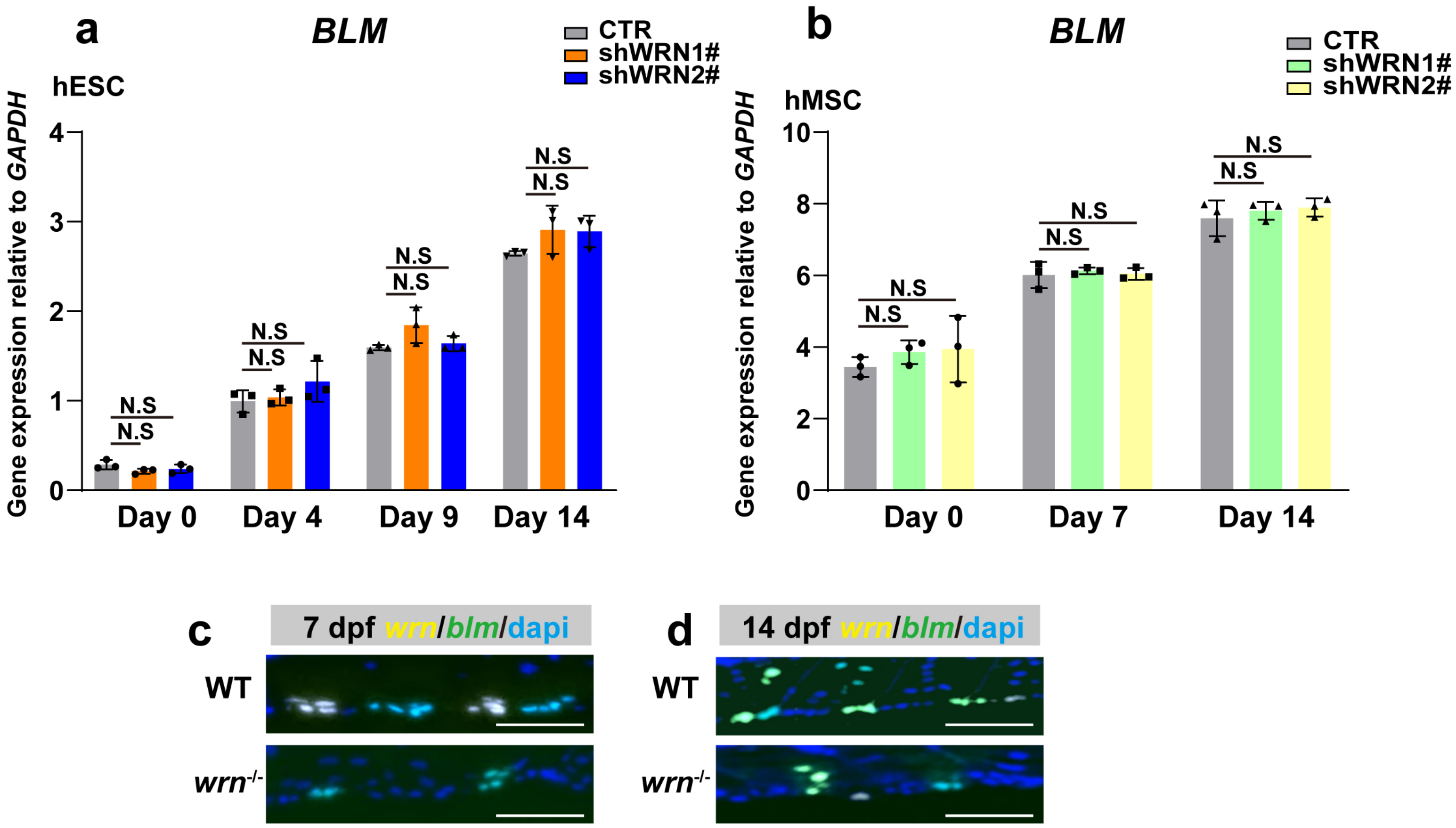

a
BLM
hESC
CTR
shWRN1#
shWRN2#
Gene expression relative to GAPDH
N.S
Day 0
Day 4
Day 9
Day 14
b
BLM
hMSC
CTR
shWRN1#
shWRN2#
Gene expression relative to GAPDH
N.S
Day 0
Day 7
Day 14
c
7 dpf wrn/blm/dapi
WT
wrn-/-
d
14 dpf wrn/blm/dapi
WT
wrn-/-

Supplementary Fig. 5. Loss of *WRN* does not affect *BLM* expression. a-b. qRT-PCR measurement of *BLM* both in hESC and hMSC on day 14. N = 3 independent biological experiments. c-d. Representative FISH analysis of three independent experiments of *wrn* and *blm* in WT and *wrn*$^{-/-}$ mutant zebrafish at 7 dpf and 14 dpf. Scale bar = 100 μm. Data are presented as the mean ± S.D. Statistical analysis was performed using two-tailed unpaired Student's t-test. *P < 0.05, **P < 0.01, ***P < 0.001.

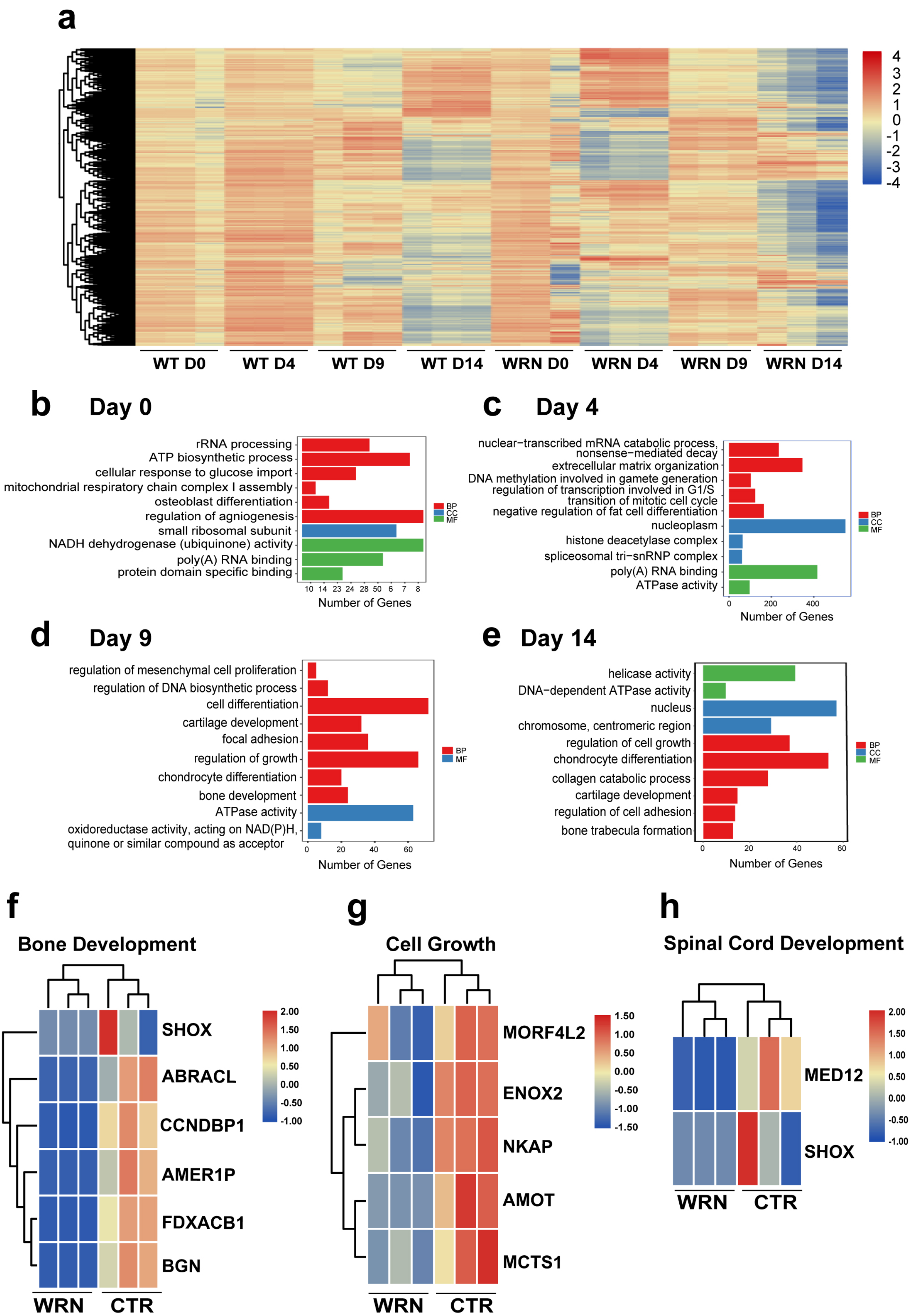
a
4
3
2
1
0
-1
-2
-3
-4
WT D0
WT D4
WT D9
WT D14
WRN D0
WRN D4
WRN D9
WRN D14
b
Day 0
rRNA processing
ATP biosynthetic process
cellular response to glucose import
mitochondrial respiratory chain complex I assembly
osteoblast differentiation
regulation of agniogenesis
small ribosomal subunit
NADH dehydrogenase (ubiquinone) activity
poly(A) RNA binding
protein domain specific binding
10 14 23 24 28 50 6 7 8
Number of Genes
BP
CC
MF
c
Day 4
nuclear−transcribed mRNA catabolic process, nonsense−mediated decay
extracellular matrix organization
DNA methylation involved in gamete generation
regulation of transcription involved in G1/S transition of mitotic cell cycle
negative regulation of fat cell differentiation
nucleoplasm
histone deacetylase complex
spliceosomal tri−snRNP complex
poly(A) RNA binding
ATPase activity
0 200 400
Number of Genes
BP
CC
MF
d
Day 9
regulation of mesenchymal cell proliferation
regulation of DNA biosynthetic process
cell differentiation
cartilage development
focal adhesion
regulation of growth
chondrocyte differentiation
bone development
ATPase activity
oxidoreductase activity, acting on NAD(P)H, quinone or similar compound as acceptor
0 20 40 60
Number of Genes
BP
MF
e
Day 14
helicase activity
DNA−dependent ATPase activity
nucleus
chromosome, centromeric region
regulation of cell growth
chondrocyte differentiation
collagen catabolic process
cartilage development
regulation of cell adhesion
bone trabecula formation
0 20 40 60
Number of Genes
BP
CC
MF
f
Bone Development
SHOX
ABRACL
CCNDBP1
AMER1P
FDXACB1
BGN
2.00
1.50
1.00
0.50
0.00
-0.50
-1.00
WRN
CTR
g
Cell Growth
MORF4L2
ENOX2
NKAP
AMOT
MCTS1
1.50
1.00
0.50
0.00
-0.50
-1.00
-1.50
WRN
CTR
h
Spinal Cord Development
MED12
SHOX
2.00
1.50
1.00
0.50
0.00
-0.50
-1.00
WRN
CTR

Supplementary Fig. 6. RNA-Seq analysis of potential target regulated by WRN in chondrocyte development homeostasis. a. The hierarchical clustering of the RNA-seq analysis results showed that all genes were significantly different expressed in 4 stages. b-e. Gene ontology (GO) term enrichment analysis of the downregulated cellular processes. The vertical coordinates represent the enriched GO terms, the horizontal green columns represent molecular functions (MF) GO terms, the red columns represent biological process (BP) GO terms and blue columns represent cellular component (CC) GO terms in 4 stages. f-h. Heatmap of representative bone development, cell growth, and spinal cord development genes.

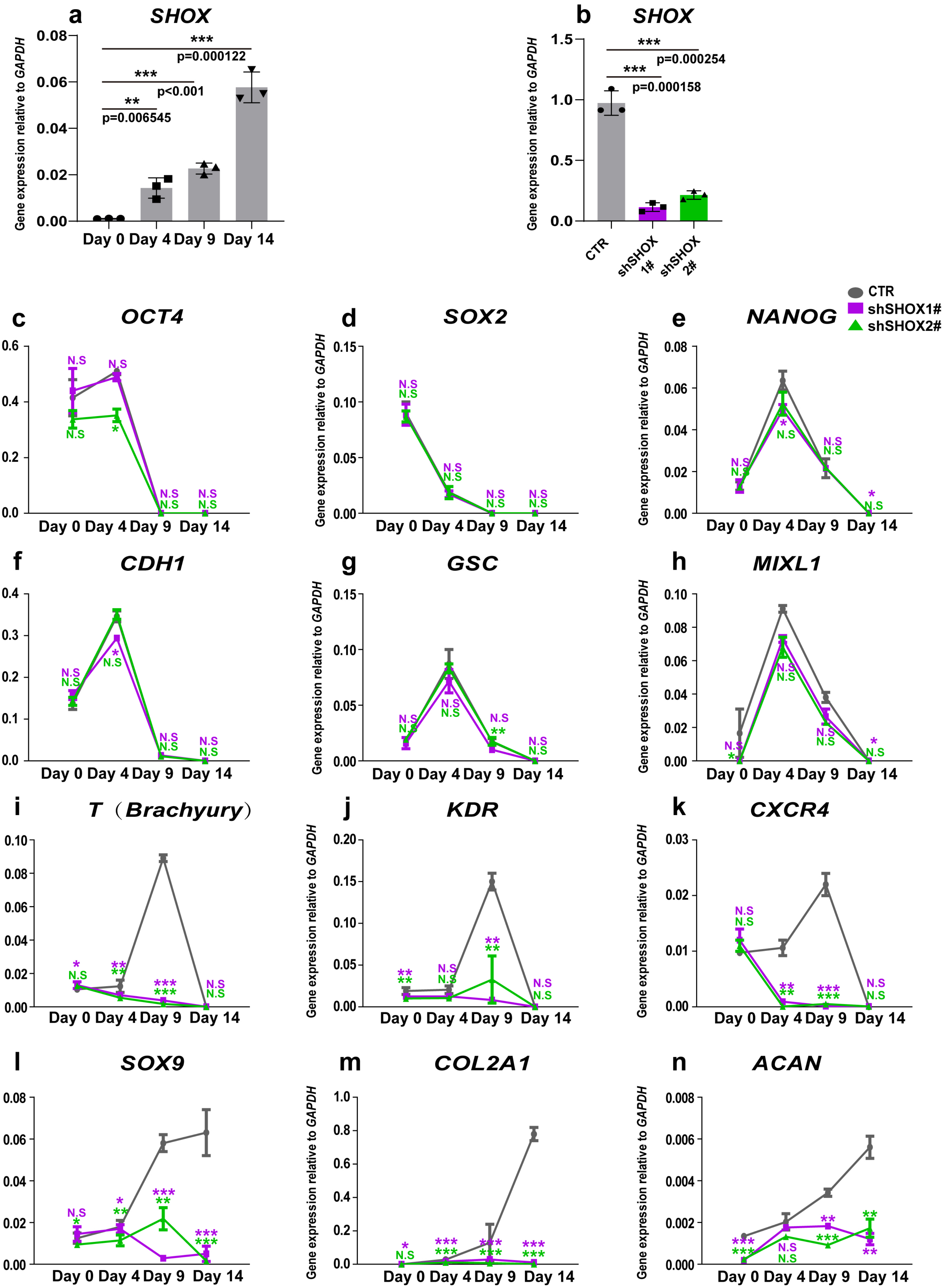
a
SHOX
b
SHOX
Gene expression relative to GAPDH
p=0.000122
p<0.001
p=0.006545
Day 0
Day 4
Day 9
Day 14
p=0.000254
p=0.000158
CTR
shSHOX 1#
shSHOX 2#
CTR
shSHOX1#
shSHOX2#
c
OCT4
d
SOX2
e
NANOG
f
CDH1
g
GSC
h
MIXL1
i
T（Brachyury）
j
KDR
k
CXCR4
l
SOX9
m
COL2A1
n
ACAN

Supplementary Fig. 7. Loss of SHOX inhibits chondrogenesis in hESCs. a. qRT-PCR measurement of the *SHOX* expression profile during chondrogenesis in the wildtype hESCs. b. qRT-PCR measurement of *WRN* knockdown efficiency. c-f. qRT-PCR measurement of hESCs at different chondrogenetic differentiation stages. c. represents genes (*NANOG*, *OCT4* and *SOX2*) related to the hESC pluripotency stage. d. represents genes (*CDH1*, *GSC* and *MIXL1*) related to the primitive streak-mesendodermal stage. e. represents genes (*T*, *KDR* and *CXCR4*) related to the mesodermal stage. f. represents genes (*SOX9*, *COL2A1* and *ACAN*) related to chondrocytes. Data are presented as the mean ± S.D. Statistical analysis was performed using two-tailed unpaired Student's t-test. N = 3 independent biological experiments. *$P < 0.05$, **$P < 0.01$, ***$P < 0.001$.

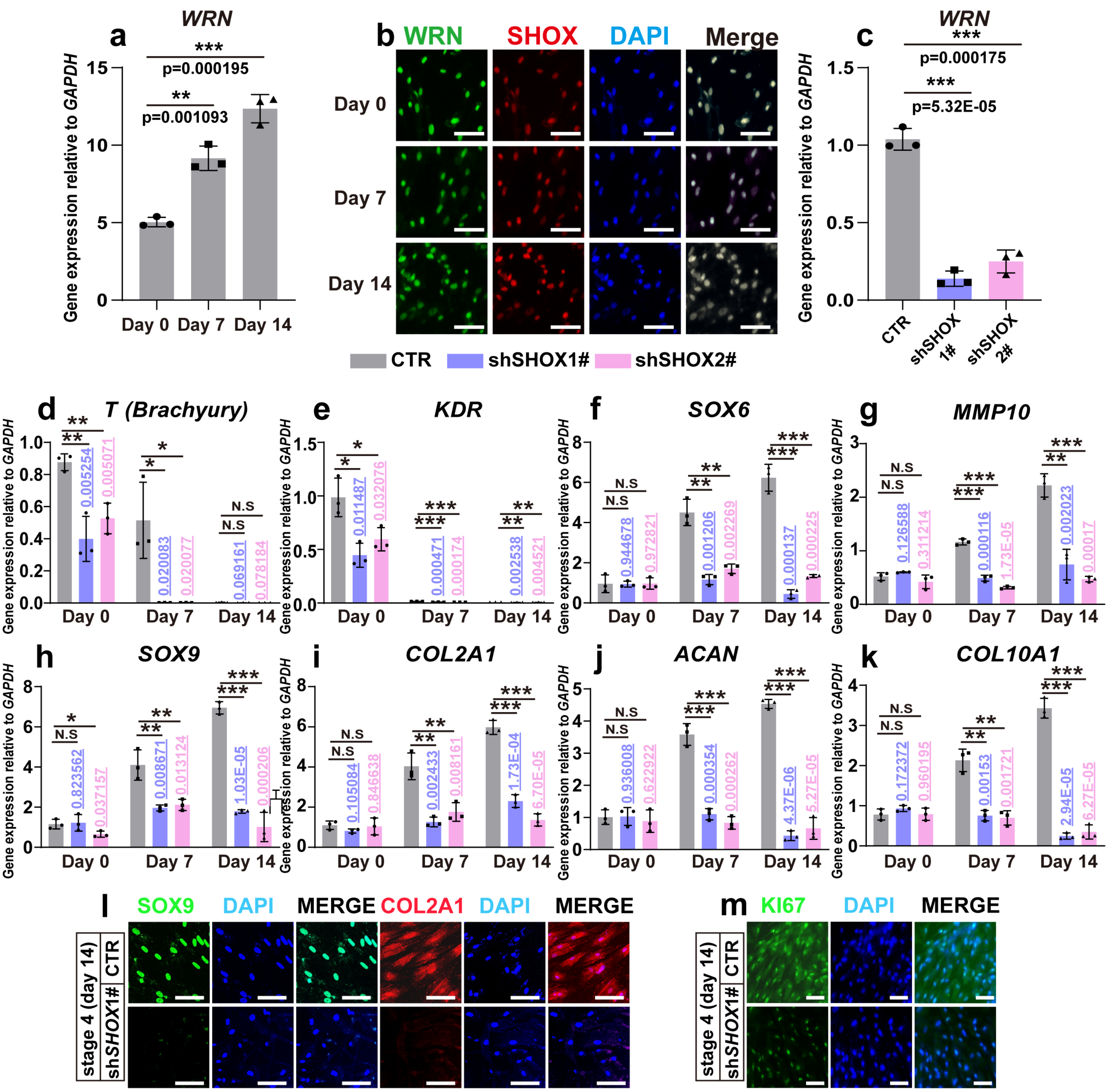

a
WRN
Gene expression relative to GAPDH
***
p=0.000195
**
p=0.001093
Day 0
Day 7
Day 14
b
WRN
SHOX
DAPI
Merge
Day 0
Day 7
Day 14
c
WRN
***
p=0.000175
***
p=5.32E-05
CTR
shSHOX 1#
shSHOX 2#
CTR
shSHOX1#
shSHOX2#
d
T (Brachyury)
e
KDR
f
SOX6
g
MMP10
h
SOX9
i
COL2A1
j
ACAN
k
COL10A1
l
SOX9
DAPI
MERGE
COL2A1
DAPI
MERGE
stage 4 (day 14)
CTR
shSHOX1#
m
KI67
DAPI
MERGE
stage 4 (day 14)
CTR
shSHOX1#

Supplementary Fig. 8. Loss of *SHOX* impairs chondrogenesis in hMSCs. a. qRT-PCR measurement of *SHOX* expression pattern profile during chondrogenesis in the wildtype hMSCs. N = 3 independent biological experiments. b. Representative immunofluorescent staining of three independent experiments of WRN and SHOX in hMSCs. Scale bar = 50 μm. c. qRT-PCR measurement of *SHOX* knockdown efficiency. N = 3 independent biological experiments. d-k. RT-PCR measurement of chondrogenic markers. N = 3 independent biological experiments. l. Representative immunofluorescent staining of three independent experiments in the CTR and shSHOX1# groups on day 14 in hMSCs. SOX9 and COL2A1 were examined. Scale bar = 50 μm. m. Representative immunofluorescent staining of three independent experiments in the CTR and shSHOX1# groups on day 14 in hMSCs. Ki67 was examined. Scale bar = 20 μm. Data are presented as the mean ± S.D. Statistical analysis was performed using two-tailed unpaired Student's t-test. *$P < 0.05$, **$P < 0.01$, ***$P < 0.001$.

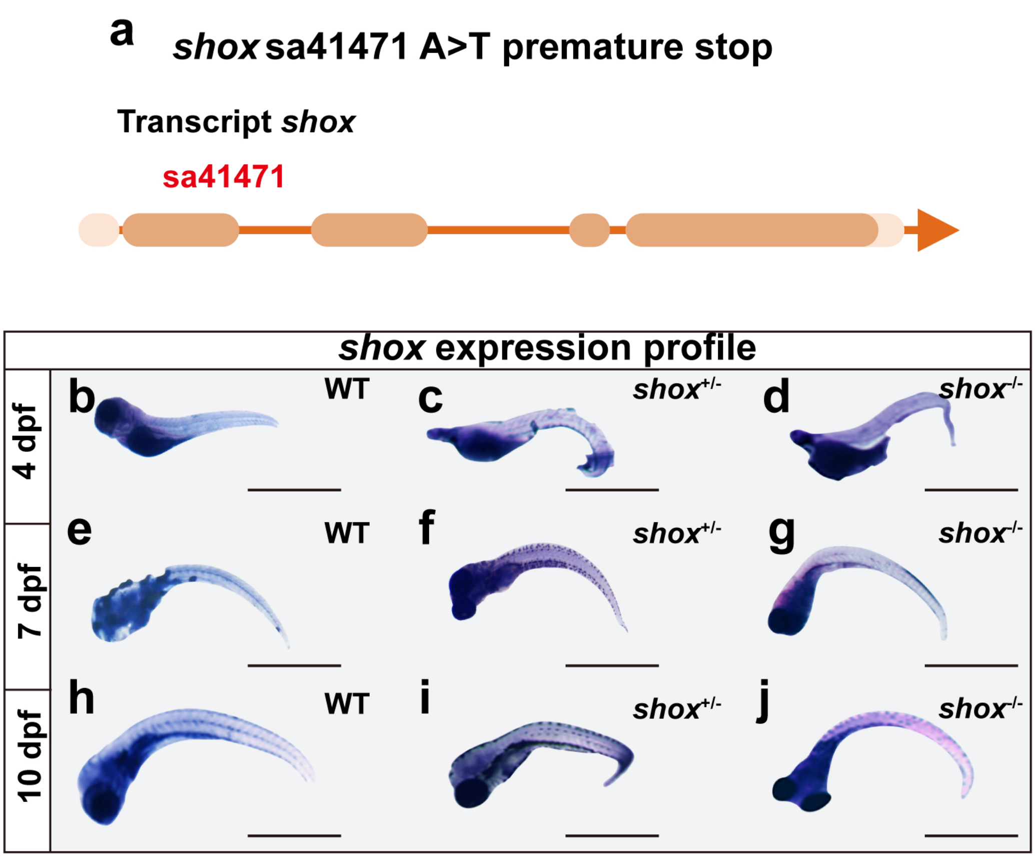
a
shox sa41471 A>T premature stop
Transcript shox
sa41471
shox expression profile
4 dpf
7 dpf
10 dpf
b
WT
c
shox+/-
d
shox-/-
e
WT
f
shox+/-
g
shox-/-
h
WT
i
shox+/-
j
shox-/-

Supplementary Fig. 9. Generation of *shox* mutant zebrafish. a. Diagram of ENU method of point mutation (*shox*^{sa41471}, A>T, premature stop) generating *shox* mutant zebrafish. Information was based on ZFIN websites (https://zfin.org/ZDB-ALT-161003-17103). b-j. Representative WISH analysis of three independent experiments of *shox* expression from 4 dpf to 10 dpf in WT, *shox*$^{-/-}$, and *shox*$^{+/-}$ zebrafish. Scale bar = 50 μm.

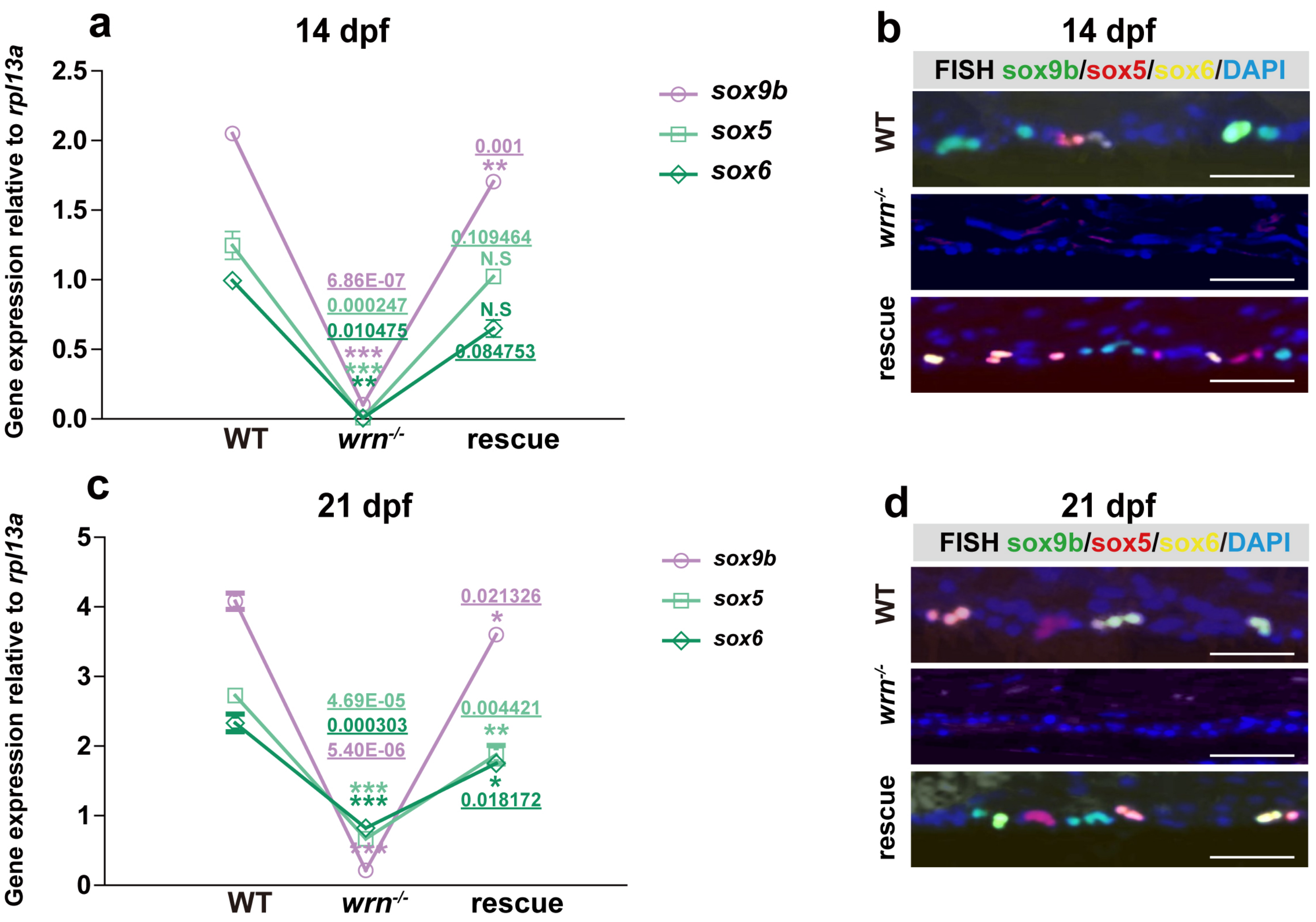
a
14 dpf
Gene expression relative to rpl13a
2.5
2.0
1.5
1.0
0.5
0.0
WT
wrn-/-
rescue
sox9b
sox5
sox6
0.001
**
0.109464
N.S
N.S
0.084753
6.86E-07
0.000247
0.010475
***
***
**
b
14 dpf
FISH sox9b/sox5/sox6/DAPI
WT
wrn-/-
rescue
c
21 dpf
Gene expression relative to rpl13a
5
4
3
2
1
0
WT
wrn-/-
rescue
sox9b
sox5
sox6
0.021326
*
4.69E-05
0.000303
5.40E-06
***
***
***
0.004421
**
*
0.018172
d
21 dpf
FISH sox9b/sox5/sox6/DAPI
WT
wrn-/-
rescue

Supplementary Fig. 10. Overexpression of *shox* promotes sox9b, sox5, and sox6 expression. a, c. qRT-PCR measurement of *sox9b*, *sox5* and *sox6* in WT zebrafish, $wrn^{-/-}$ mutant, and rescue zebrafish at 14 dpf and 21 dpf. N = 3 independent biological experiments. b, d. Representative FISH analysis of three independent experiments of *sox9b*, *sox5* and *sox6* in WT zebrafish, $wrn^{-/-}$ mutant, and rescue zebrafish at 14 dpf and 21 dpf. Scale bar = 100 μm. Data are presented as the mean ± S.D. Statistical analysis was performed using two-tailed unpaired Student's t-test. *$P < 0.05$, **$P < 0.01$, ***$P < 0.001$.

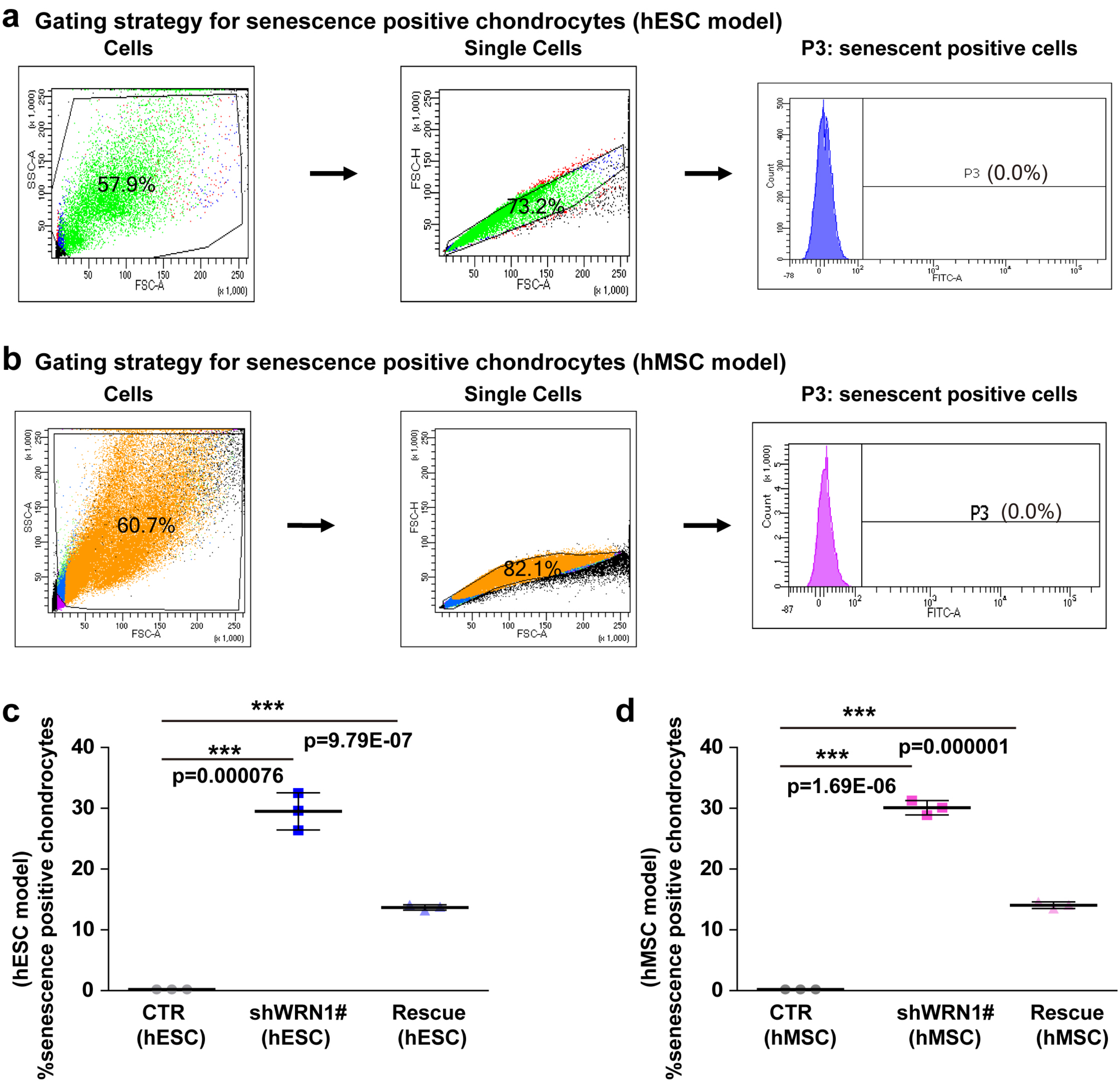
a
Gating strategy for senescence positive chondrocytes (hESC model)
Cells
Single Cells
P3: senescent positive cells
57.9%
73.2%
P3 (0.0%)
SSC-A
FSC-A
FSC-H
Count
FITC-A
b
Gating strategy for senescence positive chondrocytes (hMSC model)
Cells
Single Cells
P3: senescent positive cells
60.7%
82.1%
P3 (0.0%)
c
***
***
p=9.79E-07
p=0.000076
(hESC model)
%senescence positive chondrocytes
CTR (hESC)
shWRN1# (hESC)
Rescue (hESC)
d
***
***
p=0.000001
p=1.69E-06
(hMSC model)
%senescence positive chondrocytes
CTR (hMSC)
shWRN1# (hMSC)
Rescue (hMSC)

Supplementary Fig. 11 Flow cytometry analysis for senescence positive chondrocytes. a-b. Flow cytometry gating strategy for selecting senescence-positive chondrocytes in hESCs and hMSCs. c-d. Dot plot of three independent biological analysis of %senescence-positive chondrocytes in hESCs and hMSCs. Each dot represents a independent biological replicate. Statistical analysis was performed using two-tailed unpaired Student's t-test. Data are presented as the mean ± S.D. *$P < 0.05$, **$P < 0.01$, ***$P < 0.001$.

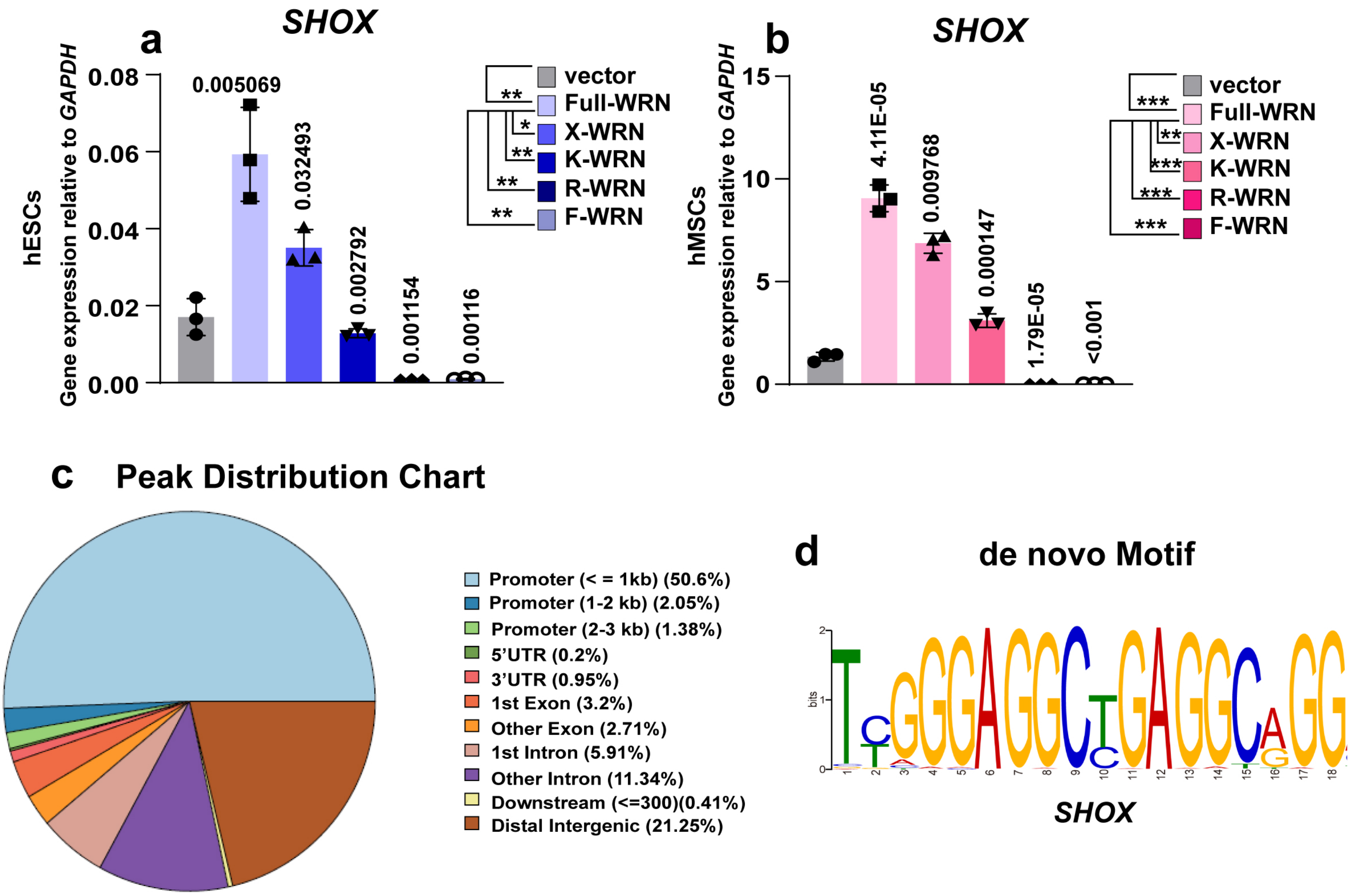

a
SHOX
hESCs
Gene expression relative to GAPDH
0.08
0.06
0.04
0.02
0.00
0.005069
0.032493
0.002792
0.001154
0.00116
vector
Full-WRN
X-WRN
K-WRN
R-WRN
F-WRN
**
*
**
**
**
b
SHOX
hMSCs
Gene expression relative to GAPDH
15
10
5
0
4.11E-05
0.009768
0.000147
1.79E-05
<0.001
vector
Full-WRN
X-WRN
K-WRN
R-WRN
F-WRN
***
**
***
***
***
c
Peak Distribution Chart
Promoter (< = 1kb) (50.6%)
Promoter (1-2 kb) (2.05%)
Promoter (2-3 kb) (1.38%)
5'UTR (0.2%)
3'UTR (0.95%)
1st Exon (3.2%)
Other Exon (2.71%)
1st Intron (5.91%)
Other Intron (11.34%)
Downstream (<=300)(0.41%)
Distal Intergenic (21.25%)
d
de novo Motif
bits
SHOX

Supplementary Fig. 12. WRN binds with SHOX in G-rich region. a-b. qRT-PCR measurement of the expression of *SHOX* on day 14 in hESCs (a) and hMSCs (b). N = 3 independent biological experiments. c. Pie chart of WRN ChIP-seq peaks distribution. d. Binding motif analysis of *WRN*-ChIP seq. Data are presented as the mean ± S.D. Statistical analysis was performed using two-tailed unpaired Student’s t-test. *$P < 0.05$, **$P < 0.01$, ***$P < 0.001$.

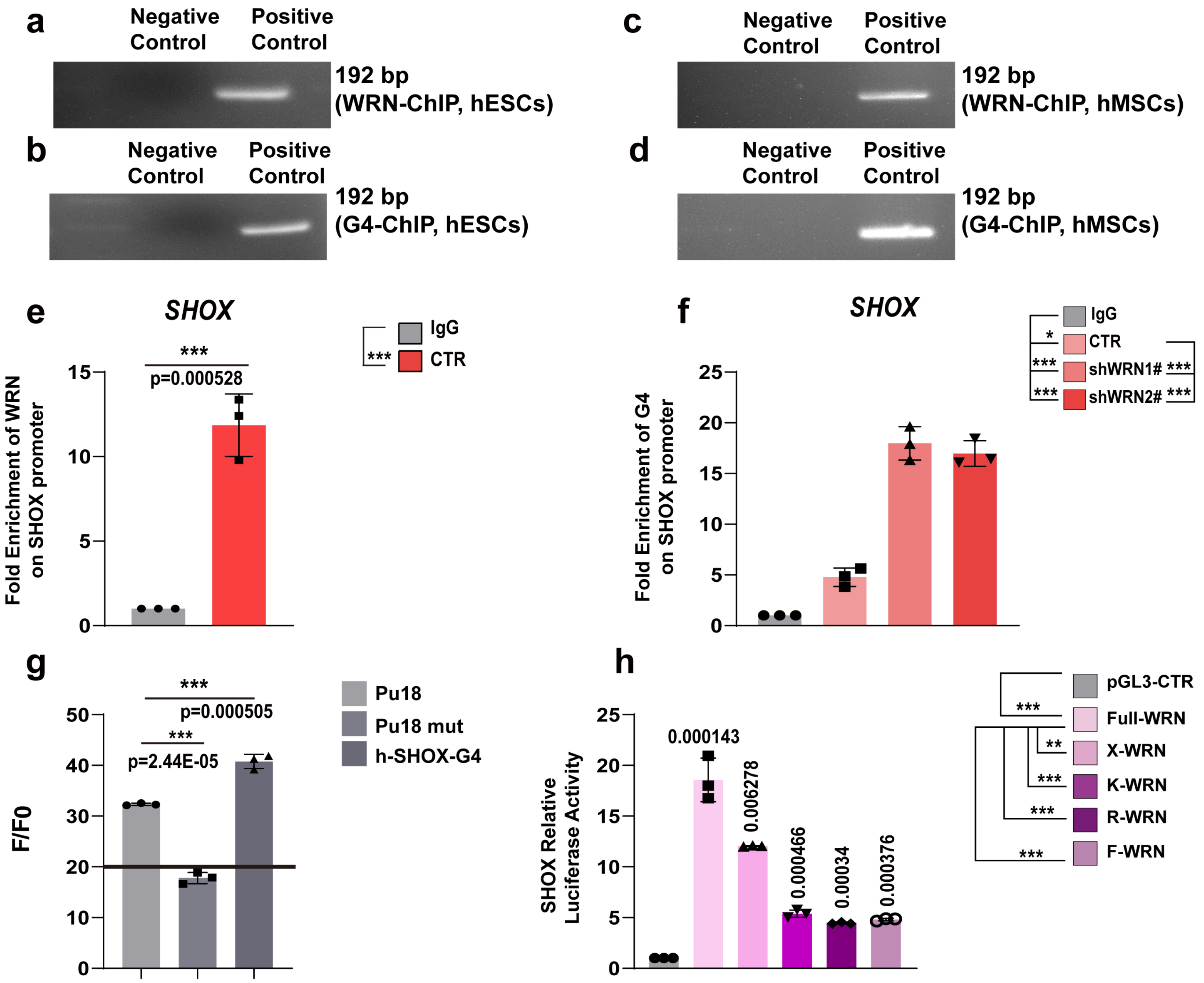

a
Negative Control
Positive Control
192 bp
(WRN-ChIP, hESCs)
b
Negative Control
Positive Control
192 bp
(G4-ChIP, hESCs)
c
Negative Control
Positive Control
192 bp
(WRN-ChIP, hMSCs)
d
Negative Control
Positive Control
192 bp
(G4-ChIP, hMSCs)
e
SHOX
***
p=0.000528
IgG
CTR
Fold Enrichment of WRN on SHOX promoter
f
SHOX
IgG
CTR
shWRN1#
shWRN2#
Fold Enrichment of G4 on SHOX promoter
g
***
p=0.000505
***
p=2.44E-05
F/F0
Pu18
Pu18 mut
h-SHOX-G4
h
0.000143
0.006278
0.000466
0.00034
0.000376
SHOX Relative Luciferase Activity
pGL3-CTR
Full-WRN
X-WRN
K-WRN
R-WRN
F-WRN

Supplementary Fig. 13. Validation of G4 existence in hMSCs. a, c. PCR gel analysis of G4 ChIP with negative and positive controls in hESCs and hMSCs. b, d. PCR gel analysis of WRN ChIP with negative and positive controls in hESCs and hMSCs. e. ChIP-qPCR for WRN-SHOX. f. ChIP-qPCR for G4-SHOX. g. Bar graph of fluorescence enhancement (F/F_0) in Pu18, Pu18 mut, and h-SHOX-G4 groups. h. Luciferase assay of the *SHOX* transcription activity. N = 3 independent biological experiments. Data are presented as the mean ± S.D. Statistical analysis was performed using two-tailed unpaired Student's t-test. **P* < 0.05, ***P* < 0.01, ****P* < 0.001.

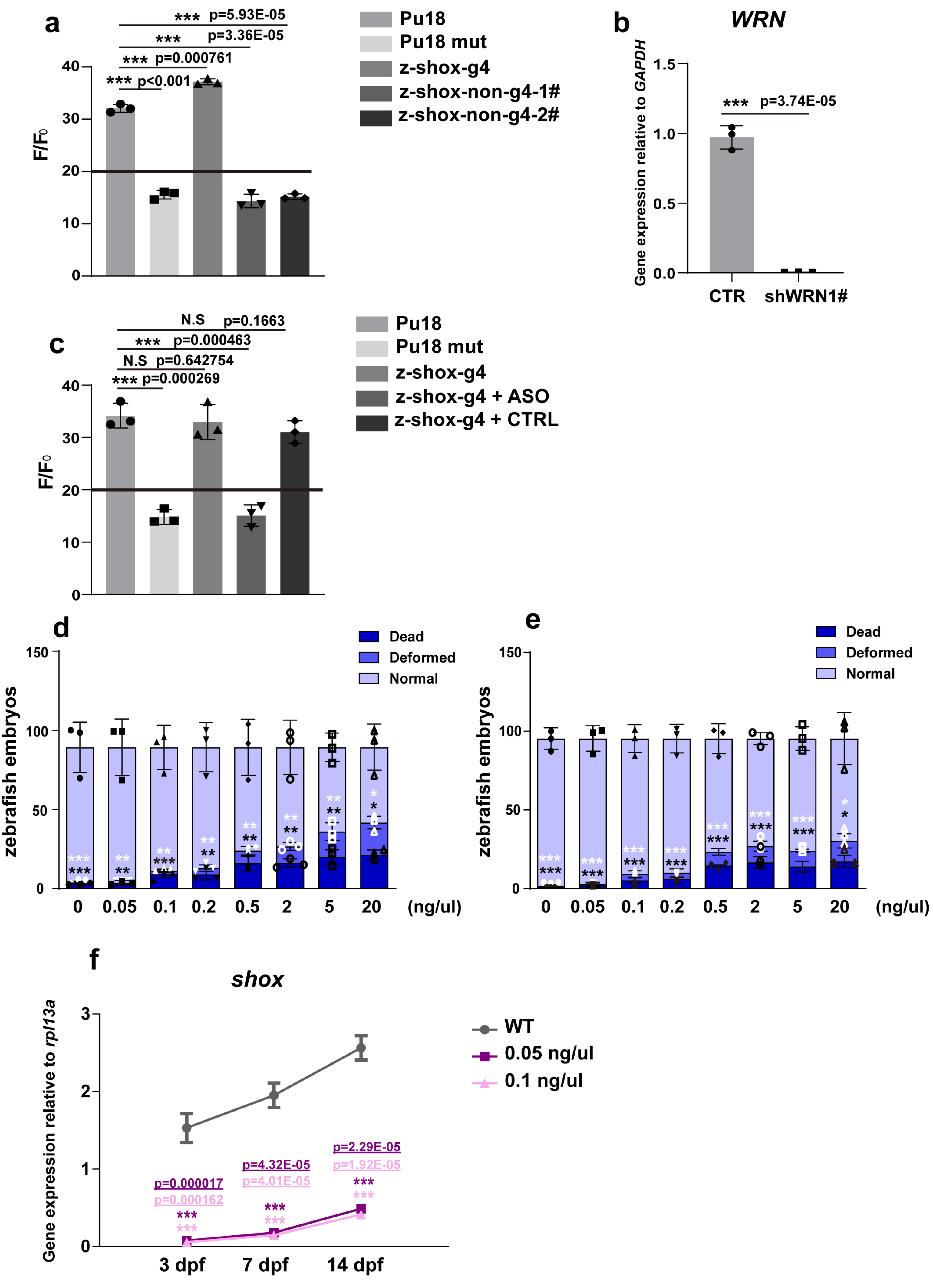
a
*** p=5.93E-05
*** p=3.36E-05
*** p=0.000761
*** p<0.001
F/F0
Pu18
Pu18 mut
z-shox-g4
z-shox-non-g4-1#
z-shox-non-g4-2#
b
WRN
Gene expression relative to GAPDH
*** p=3.74E-05
CTR
shWRN1#
c
N.S p=0.1663
*** p=0.000463
N.S p=0.642754
*** p=0.000269
F/F0
Pu18
Pu18 mut
z-shox-g4
z-shox-g4 + ASO
z-shox-g4 + CTRL
d
zebrafish embryos
Dead
Deformed
Normal
0
0.05
0.1
0.2
0.5
2
5
20
(ng/ul)
e
zebrafish embryos
Dead
Deformed
Normal
0
0.05
0.1
0.2
0.5
2
5
20
(ng/ul)
f
shox
Gene expression relative to rpl13a
WT
0.05 ng/ul
0.1 ng/ul
p=0.000017
p=0.000162
p=4.32E-05
p=4.01E-05
p=2.29E-05
p=1.92E-05
3 dpf
7 dpf
14 dpf

Supplementary Fig. 14. Validation of G4 existence in zebrafish. a. Bar graph of fluorescence enhancement (F/F_0) in Pu18, Pu18 mut, z-shox-g4, z-shox-non-g4-1#, and z-shox-non-g4-2# groups. b. qRT-PCR measurement of *SHOX* knockdown efficiency in 293T cells. c. Bar graph of fluorescence enhancement (F/F_0) in Pu18, Pu18 mut, z-shox-g4, z-shox-g4+ASO, and z-shox-g4+CTRL groups. d-e. Stacked bar graphs of zebrafish embryos in dead, deformd, and normal status with different G4 stabilizer concentration at 24 hpf (d) and 48 hpf (e). f. qRT-PCR measurement of *shox*. Data are presented as the mean ± S.D. Statistical analysis was performed using two-tailed unpaired Student's t-test. *P < 0.05, **P < 0.01, ***P < 0.001.

Table 1.

qPCR	Primer sequences (5’---3’)
h-OCT4 F	AGACCATCTGCCGCTTTGAG
h-OCT4 R	GCAAGGGCCGCAGCTT
h-SOX2 F	TGGTCCTGCATCATGCTGTAG
h-SOX2 R	AACCAGCGCATGGACAGTTAC
h-NANOG F	GGCTCTGTTTTGCTATATCCCCTAA
h-NANOG R	CATTACGATGCAGCAAATACAAGA
h-CDH1 F	GCTGGACCGAGAGAGTTTCC
h-CDH1 R	CAAAATCCAAGCCCGTGGTG
h-GSC F	GATGCTGCCCTACATGAACGT
h-GSC R	GACAGTGCAGCTGGTTGAGAAG
h-MIXL1 F	AAGCCCCAGCTGCCTGTT
h-MIXL1 R	CCCTCCAACCCCGTTTG
h-T(Brachyury) F	GGGTCCACAGCGCATGAT
h-T(Brachyury) R	TGATAAGCAGTCACCGCTATGAA
h-KDR F	GTAACCCGGAGTGACCAAGG
h-KDR R	AACCAAGGTACTTCGCAGGG
h-CXCR4 F	CGCCTGTTGGCTGCCTTA
h-CXCR4 R	ACCCTTGCTTGATGATTTCCA
h-SOX9 F	GACTTCCGCGACGTGGAC
h-SOX9 R	GTTGGGCGGCAGGTACTG
h-COL2A1 F	GGCAATAGCAGGTTCACGTACA
h-COL2A1 R	CGATAACAGTCTTGCCCCACTT
h-ACAN F	TCGAGGACAGCGAGGCC
h-ACAN R	TCGAGGGTGTAGCGTGTAGAGA
h-SOX6 F	GCAGTGATCAACATGTGGCCT
h-SOX6 R	CGCTGTCCCAGTCAGCATCT
h-WRN F	GCATGCACTTATCCCAAGCG
h-WRN R	GTTGAAGTCCGCTGTCAGGA
h-SHOX F	CAACATGGGAGCCTTACGGA

h-SHOX R	CGATGCTGGAATTCTTGCTGT
h-BLM F	GAGTCTGCGTGCGAGGATTA
h-BLM R	AGTGTTCTGGCTGAGTGACG
h-P16 F	GAAGGTCCCTCAGACATCCCC
h-P16 R	CCCTGTAGGACCTTCGGTGAC
h-P53 F	CCCCTCCTGGCCCCTGTCATCTTC
h-P53 R	GCAGCGCCTCACAACCTCCGTCAT
h-BCL2 F	CATGCCAAGAGGGAAACACCAGAA
h-BCL2 R	GTGCTTTGCATTCTTGGATGAGGG
h-CASPASE 8 F	ACAAGGGCATCATCTATGGCTCTGA
h-CASPASE 8 R	CCAGTGAAGTAAGAGGTCAGCTCAT
h-GAPDH F	ATGGGGAAGGTGAAGGTCG
h-GAPDH R	TAAAAGCAGCCCTGGTGACC
z-sox9a F	TGAACGAGGTGGAAAAGCGT
z-sox9a R	CTTTGAAGATGGCGTTGGGC
z-col2a1a F	CAGTCCCACTTCACCCCTTAC
z-col2a1a R	ACTCTGGGCAATCATGGGAG
z-col1a1a F	ACTTGCTTAGACCTGCGCTT
z-col1a1a R	CCAGGGGGATTTTACACGCT
z-col10a1a F	ATGGAACTACGAGTAGTAAGCATTCTT
z-col10a1a R	TGGCAGACCTTCACCATCTTGTCCTG
z-sox9b F	GCAAAACACTCGGCAAACTCT
z-sox9b R	CTGAGCCGCTCTTCACTGATT
z-sox5 F	GACCCGTGAACGCCTTT
z-sox5 R	CCGCATTTCTTGTCTGCGATT
z-sox6 F	GACTGAACACTGGCGAGCTGTTA
z-sox6 R	GATCTGCTCTTGCTGTTGGC
z-wrn F	CATCTGTCCAAACACACTCCAGC
z-wrn R	GCGAGCATCTGATACCTGCC
z-shox F	CCGCCCTTTGGATTACCGAT
z-shox R	CACGGCCTTTTCACAGGCTT
z-rpl13α F	TCTGGAGGACTGTAAGAGGTATGC
z-rpl13α R	AGACGCACAATCTTGAGAGCAG

WISH	
z-sox9a F	GCGCTTTGCTTTCGCAGACACCA
z-sox9a R	CAAAGTTTTCCCAGAGTTTTGC
z-col2a1a F	AGGCCCAGAAGCACGTGTGGT
z-col2a1a R	GCTCCCGTCAAGAGGAGGCGCTGC
z-col1a1a F	TATGGCAGCGAGGGCTCCAAGCCT
z-col1a1a R	TCACTTGTATCATTGATGTTTCT
z-col10a1a F	GTCTGGCTCATACCACAAGGAT
z-col10a1a R	TTCCCCATCACGGCCCAGGT
z-wrn F	TGGCGTTCCTCAGTATTTCTGA
z-wrn R	CCCACGCCAACTTTCATGATGTTCT
z-shox F	AACAGCAGGAGTGATCTAA
z-shox R	TCAGAGTCCCAGCGCCTCGGCGT
FISH	
z-sox9a F	GCGCTTTGCTTTCGCAGACACCA
z-sox9a R	CAAAGTTTTCCCAGAGTTTTGC
z-col2a1a F	AGGCCCAGAAGCACGTGTGGT
z-col2a1a R	GCTCCCGTCAAGAGGAGGCGCTGC
z-col1a1a F	TATGGCAGCGAGGGCTCCAAGCCT
z-col1a1a R	TCACTTGTATCATTGATGTTTCT
z-col10a1a F	GTCTGGCTCATACCACAAGGAT
z-col10a1a R	TTCCCCATCACGGCCCAGGT
z-sox9b F	TTTACTTCAGCCAAACACACACTT
z-sox9b R	TTGCCGAGTGTTTTGCTGAGCTC
z-sox5 F	GGGAGCACCAGGCAGTGTGTGCG
z-sox5 R	CCCATGGCCAATAGCTTGTCCT
z-sox6 F	CTTTCTGCGTAGCAGCTTTTTACT
z-sox6 R	TGCTGCAGAAGTTGCTGCTGTTGTCT
z-wrn F	TGGCGTTCCTCAGTATTTCTGA
z-wrn R	CCCACGCCAACTTTCATGATGTTCT
z-shox F	AACAGCAGGAGTGATCTAA
z-shox R	TCAGAGTCCCAGCGCCTCGGCGT
z-p53 F	CTGTAACTAGGGGAATCCCCA

z-p53 R	ATCCGGGGTTCGCTCATGATGGGG
z-p16 F	ACGAAAAATTATTTTAAGACACA
z-p16 R	GCTATGCAGTGATGCAGTCTT
ChIP-qPCR	
G4 ChIP-qPCR F	TCCGGGACCCTCTGCAGCC
G4 ChIP-qPCR R	TTGCACCCCCGTCATGCAGCCCA
WRN ChIP-qPCR F	TCCGGGACCCTCTGCAGCC
WRN ChIP-qPCR R	TTGCACCCCCGTCATGCAGCCCA
Oligonucleotides	
Shox-g4-ASO	GGCGATGGGGGCCGGGCTGAGG
h-G4 F	GGGAGTTGGGGAGGAATTCAGGGAAGGGGG
h-G4 R	ACCCCCTTCCCTGAATTCCTCCCCAACTCCC
z-g4 F	GGCGATGGGGGCCGGGCTGAGG
z-g4 R	CCTCAGCCCGGCCCCCATCGCC
z-non-g4-1# F	CTGTAATCAGCCATGGAAGAAC
z-non-g4-1# R	GTTCTTCCATGGCTGATTACAG
z-non-g4-2# F	CCTAATTAATTATAATTAAAT
z-non-g4-2# R	ATTTAATTATAATTAATTAGG
CTR (shNC)	CCGGCAACAAGATGAAGAGCACCAACTCGAGTTGGTGCTCTTCATCTTGTTGTTTT
shWRN-1#	AAAAGGAAACTTCTACGTGACTTTTGGATCCAAAAGTCACGTAGAAGTTTCC
shWRN-2#	AAAAGCAGTCTCAGGAAGAATATTTGGATCCAAATATTCTTCCTGAGACTGC
shSHOX-1#	AAAAGGCAGAAGGGATTTATGAATTGGATCCAATTCATAAATCCCTTCTGCC
shSHOX-2#	AAAAGGAAGATCCTTAGAGTCTATTGGATCCAATAGACTCTAAGGATCTTCC

WRN mutations	
X-WRN-F	TTTGACATGGCGTGGCCACCATTATAC
X-WRN-R	TCCCACCACATCCCCATC
K-WRN-F	GGATATGGAATGAGTTTGTGCTTC
K-WRN-R	AGTTGCCATGACAGCAAC
R-WRN-F	TAATTCTCAGGCTCTTGCCGATCAATATCG
R-WRN-R	GATCCTCGGAGAAATAAAATTG
F-WRN-F	GTATAACAAAGCTATGAAGATTTGCGCC
F-WRN-R	CGAGAAACTTCTACCAAG
h: human	
z: zebrafish	

Table 2

Protein	Antibody
Collagen II	Santa Cruz, sc-518017
SOX9	R&D systems, AF3075
WRN	Sigma, W0393
SHOX	Thermo Fisher Invitrogen, PA5-65140
G4 (clone 1H6)	Merck, MABE1126
gamma H2AX	Gentex, GTX127342
Alexa Fluor 488-conjuated anti-mouse IgG	Invitrogen, A28175
Alexa Fluor 488-conjuated anti-rabbit IgG	Invitrogen, A11008
Alexa Fluor 594-conjuated anti-rabbit IgG	Invitrogen, A11037
