# Supplementary material for: WRN promotes bone development and growth by unwinding SHOX-G-quadruplexes via its helicase activity in Werner Syndrome
Source: Nat Commun. 2022 Sep 16;13:5456. doi: 10.1038/s41467-022-33012-6 (PMC9481537; doi:10.1038/s41467-022-33012-6)
Supplement: Supplementary file 3 — Reporting Summary [file 41467_2022_33012_MOESM3_ESM.pdf]

nature portfolio

Corresponding author(s): Wai-Yee Chan

Last updated by author(s): Jul 30, 2022

Reporting Summary

Nature Portfolio wishes to improve the reproducibility of the work that we publish. This form provides structure for consistency and transparency in reporting. For further information on Nature Portfolio policies, see our Editorial Policies and the Editorial Policy Checklist.

Statistics

For all statistical analyses, confirm that the following items are present in the figure legend, table legend, main text, or Methods section.

n/a	Confirmed	
☐	☒	The exact sample size (*n*) for each experimental group/condition, given as a discrete number and unit of measurement
☐	☒	A statement on whether measurements were taken from distinct samples or whether the same sample was measured repeatedly
☐	☒	The statistical test(s) used AND whether they are one- or two-sided<br>*Only common tests should be described solely by name; describe more complex techniques in the Methods section.*
☒	☐	A description of all covariates tested
☒	☐	A description of any assumptions or corrections, such as tests of normality and adjustment for multiple comparisons
☐	☒	A full description of the statistical parameters including central tendency (e.g. means) or other basic estimates (e.g. regression coefficient) AND variation (e.g. standard deviation) or associated estimates of uncertainty (e.g. confidence intervals)
☐	☒	For null hypothesis testing, the test statistic (e.g. *F*, *t*, *r*) with confidence intervals, effect sizes, degrees of freedom and *P* value noted<br>*Give P values as exact values whenever suitable.*
☒	☐	For Bayesian analysis, information on the choice of priors and Markov chain Monte Carlo settings
☒	☐	For hierarchical and complex designs, identification of the appropriate level for tests and full reporting of outcomes
☒	☐	Estimates of effect sizes (e.g. Cohen's *d*, Pearson's *r*), indicating how they were calculated

Our web collection on statistics for biologists contains articles on many of the points above.

Software and code

Policy information about availability of computer code

Data collection	Guadruplex structure prediction was acquired by QGPS mapper website (https://bioinformatics.ramapo.edu/QGRS/index.php)<br>Cellular fluorescent Images were acquired by Leica FV 1200 confocol microscope or Nikon Eclipse Ts2 microscope.<br>Whole-mount in situ hybridization images and calcein green images were acquired by Olympus SZX16 microscope.<br>Masson trichrome staining images were acquired by Nikon Ni-U Eclipse Upright Microscope (CTS).<br>Fluorescent in situ hybridization images were acquired by Leica TCS SP8 confocal microscope.<br>ThT fluorescence assay was acquired by SpectraMax M3 microplate reader.<br>Flow cytometry data was acquired by BD FACSAria Fusion.<br>Quantitative real-time PCR data was acquired by QuantStudio Real-time PCR.<br>Nikon SMZ800 stereomicroscope was used for microinjection.
Data analysis	Image J (version 1. 52)was used for immunofluorescence analysis.<br>GraphPad Prism (version 8.0) was used for statistic analysis.<br>QuantStudio Real-time PCR software (version 1. 7. 2) was used for real-time pcr analysis.<br>BD FACS DIVA software (version 9.3) was usedfor flow cytometry analysis.<br>MACS (version 2.1.2) was used for ChIP peak calling.<br>Olympus cellSens Standard (version 1. 18) was used for calcein green staining analysis.<br>SoftMax Pro (version 7.0) was used for ThT fluorescence intensity analysis.<br>NIS-Elements BR software (version 5.20.02) was used for Masson trichrome staining analysis.

For manuscripts utilizing custom algorithms or software that are central to the research but not yet described in published literature, software must be made available to editors and reviewers. We strongly encourage code deposition in a community repository (e.g. GitHub). See the Nature Portfolio guidelines for submitting code & software for further information.

Data

Policy information about availability of data

All manuscripts must include a data availability statement. This statement should provide the following information, where applicable:

- Accession codes, unique identifiers, or web links for publicly available datasets
- A description of any restrictions on data availability
- For clinical datasets or third party data, please ensure that the statement adheres to our policy

All source data supporting the findings of this study are available in the paper, and/or from the corresponding author on a reasonable request. All sequencing data that support the findings of this study have been deposited in the National Center for Biotechnology Information (NCBI) Sequence Read Archive (SRA) under accession numbers GSE206214 (https://www.ncbi.nlm.nih.gov/geo/query/acc.cgi?acc=GSE206214) (GSE206213 for RNA-seq and GSE 206210 for ChIP-seq).

Field-specific reporting

Please select the one below that is the best fit for your research. If you are not sure, read the appropriate sections before making your selection.

☒ Life sciences ☐ Behavioural & social sciences ☐ Ecological, evolutionary & environmental sciences

For a reference copy of the document with all sections, see nature.com/documents/nr-reporting-summary-flat.pdf

Life sciences study design

All studies must disclose on these points even when the disclosure is negative.

Sample size	The sample sizes were determined based on our preliminary studies in our laboratory or in similarly published research (PMID: 24212092; PMID: 32868902; PMID: 28530658). The samples were enough for study and showed significant statistical difference in the replicated independent experiments. Please see figure legends for each experiment.
Data exclusions	No data were excluded from the analysis.
Replication	All experiments were replicated in at least three independent biological replicates. All attempts were successful.
Randomization	Microscopic images were acquired randomized on any given slide. Zebrafish samples used were randomly selected for each treatment group. For in vivo experiments, all cells were maintained in the same environment and were randomly assigned to each treatment group.
Blinding	The investigators were blinded to allocation during experiments and outcome assessment.

Reporting for specific materials, systems and methods

We require information from authors about some types of materials, experimental systems and methods used in many studies. Here, indicate whether each material, system or method listed is relevant to your study. If you are not sure if a list item applies to your research, read the appropriate section before selecting a response.

Materials & experimental systems

n/a	Involved in the study	
☐	☒	Antibodies
☐	☒	Eukaryotic cell lines
☒	☐	Palaeontology and archaeology
☐	☒	Animals and other organisms
☒	☐	Human research participants
☒	☐	Clinical data
☒	☐	Dual use research of concern

Methods

n/a	Involved in the study	
☐	☒	ChIP-seq
☐	☒	Flow cytometry
☒	☐	MRI-based neuroimaging

Antibodies

Antibodies used

SOX 9 (R&D systems), Cat#AF3075
COL2A1 (Santa Cruz), Cat#sc-518017
WRN (Sigma), Cat#W0393
SHOX (Thermo Fisher Invitrogen), Cat#PA5-65140
G4 (clone 1H6) (Merck), Cat#MABE1126
KI-67 (BD, Biosciences), Cat#556003
gamma-H2AX (Gentex), Cat#GTX127342
Alexa Fluor 488-conjugated anti-mouse IgG (Invitrogen), Cat#A28175

	Alexa Fluor 488-conjugated anti-rabbit IgG (Invitrogen), Cat#A-11008<br>Alexa Fluor 594-conjugated anti-rabbit IgG (Invitrogen), Cat#A-11037
Validation	All antibodies were well-recognized clones used in the research and acquired from reputable vendors. Each antibody used in this study has been validated for its utilized manufacture and all the related product information is available from manufactures' website.<br>Detailed is as following:<br>SOX9 (https://www.rndsystems.com/cn/products/human-sox9-antibody_af3075)<br>COL2A1 (https://www.scbt.com/p/col2a1-antibody-b-1?gclid=EAIaIQobChMI57nw-o288wIVe9xMAh20Gw1sEAAYBCAAEgK_GfD_BwE)<br>WRN (https://www.sigmaaldrich.com/HK/en/product/sigma/w0393)<br>SHOX (https://www.thermofisher.com/antibody/product/SHOX-Antibody-Polyclonal/PA5-65140)<br>G4 (clone 1H6) (https://www.merckmillipore.com/HK/en/product/Anti-DNA-G-quadruplex-G4-Antibody-clone-1H6,MM_NF-MABE1126?ReferrerURL=https%3A%2F%2Fwww.google.com%2F)<br>KI-67 (https://www.bdbiosciences.com/en-us/products/reagents/flow-cytometry-reagents/research-reagents/single-color-antibodies-ruo/purified-mouse-anti-ki-67.556003)<br>gammaH2AX (https://www.genetex.com/Product/Detail/Histone-H2A-XS139ph-phospho-Ser139-antibody/GTX127342#datasheet)<br>Alexa Fluor 488-conjugated anti-mouse IgG (https://www.thermofisher.com/antibody/product/Goat-anti-Mouse-IgG-H-L-Secondary-Antibody-Recombinant-Polyclonal/A28175)<br>Alexa Fluor 488-conjugated anti-rabbit IgG (https://www.thermofisher.com/antibody/product/Goat-anti-Rabbit-IgG-H-L-Cross-Adsorbed-Secondary-Antibody-Polyclonal/A-11008)<br>Alexa Fluor 594-conjugated anti-rabbit IgG (https://www.thermofisher.com/antibody/product/Goat-anti-Rabbit-IgG-H-L-Highly-Cross-Adsorbed-Secondary-Antibody-Polyclonal/A-11037)<br><br>The antibody dilution ration has been specified in methods.

Eukaryotic cell lines

Policy information about cell lines

Cell line source(s)	H1 human embryonic stem cells (hESCs), human mesenchymal stem cells (Lonza Bioscience, Cat.PT-2501)
Authentication	Cell lines were not authenticated.
Mycoplasma contamination	hMSCs were tested negative for mycoplasma contamination.
Commonly misidentified lines (See ICLAC register)	No commonly misidentified lines were used in this study.

Animals and other organisms

Policy information about studies involving animals; ARRIVE guidelines recommended for reporting animal research

Laboratory animals	wrn(sa34829) and shox (sa41471) zebrafish line were acquired from the Zebrafish Information Network (ZFIN,https://zfin.org/). 3 dpf, 4 dpf, 10 dpf, 14 dpf, and 40 dpf timepoints were selected for study. Both male and female fish were tested, and we treated them equally at the same time.
Wild animals	No wild animals were used in the study.
Field-collected samples	No field collected samples were used in the study.
Ethics oversight	Zebrafish holding and experimentation were performed according to protocols approved by the Animal Experimentation Ethics Committee (AEEC, Ref No. 20-200-MIS) of the Chinese University of Hong Kong.

Note that full information on the approval of the study protocol must also be provided in the manuscript.

ChIP-seq

Data deposition

☒ Confirm that both raw and final processed data have been deposited in a public database such as GEO.

☒ Confirm that you have deposited or provided access to graph files (e.g. BED files) for the called peaks.

Data access links<br>*May remain private before publication.*	https://www.ncbi.nlm.nih.gov/geo/query/acc.cgi?acc=GSE206214
Files in database submission	Input2. fq.gz<br>Input3.fq.gz<br>WRN1.fq.gz<br>WRN2.fq.gz<br>WRN3.fq.gz

WRN1.bam.bw
WRN2.bam.bw
WRN3.bam.bw

WRN1.bam_summits.bed
WRN2.bam_summits.bed
WRN3.bam_summits.bed

Genome browser session (e.g. UCSC): no longer applicable

Methodology

Replicates: Two biological replicates were performed for all sequencing experiments.

Sequencing depth: An average 6G data output per sample (paired-end sequencing).

Antibodies: WRN (Sigma), W0393

Peak calling parameters: Peak calling was performed using MASC software by Novogene Company (Beijing, China).

Data quality: Data quality for each replicate was confirmed by FastQC in Novogene Company (Beijing, China).

Software: hg39 as reference genome.
MASC (v.2.1.2) for peak calling.

Flow Cytometry

Plots

Confirm that:

☒ The axis labels state the marker and fluorochrome used (e.g. CD4-FITC).

☒ The axis scales are clearly visible. Include numbers along axes only for bottom left plot of group (a 'group' is an analysis of identical markers).

☒ All plots are contour plots with outliers or pseudocolor plots.

☒ A numerical value for number of cells or percentage (with statistics) is provided.

Methodology

Sample preparation: H1 human embryonic stem cells (hESCs) and human mesenchymal stem cells (hMSCs) were differentiated towards chondrocytes. The CTR-hESCs, shWRN1#hESCs, rescue-hESCs, CTR-hMSCS, shWRN1#, and rescue-hMSCs were collected on day 14. Trypsinized cells were collected for flow cytometry.

Instrument: BD FACSAria™Fusion (BD Biosciences) was applied for flow cytometry.

Software: BD FACSDiva Software v9.3 was applied to acquire flow cytometry data.

Cell population abundance: Populations were identified as described in the Methods.

Gating strategy: The gating strategy for floe cytometry analysis was detailed in results 8. and supplementary 11 section.
We set the preliminary FSC-A vs SSC-A gate strategy based on the cell size and complexity to exclude debris and other events of non-interest, then we set the gate for senescence-positive hESC populations or hMSC populations based on negative hESCs or hMSCs (control, CTR). The percentage of senescence-positive cells are: 0.0% (CTR, hESCs), 29.6% (shWRN1#, hESCs), 13.1% (rescue, hESCs), 0.2% (CTR, hMSCs), 30.1% (shWRN1#, hMSCs), and 14.1% (rescue, hMSCs).

☒ Tick this box to confirm that a figure exemplifying the gating strategy is provided in the Supplementary Information.
