# Supplementary figures and images for: WRN promotes bone development and growth by unwinding SHOX-G-quadruplexes via its helicase activity in Werner Syndrome
Source: Nat Commun. 2022 Sep 16;13:5456. doi: 10.1038/s41467-022-33012-6 (PMC9481537; doi:10.1038/s41467-022-33012-6)

**Supplementary Fig. 13 a-d**

**uncropped DNA gels**

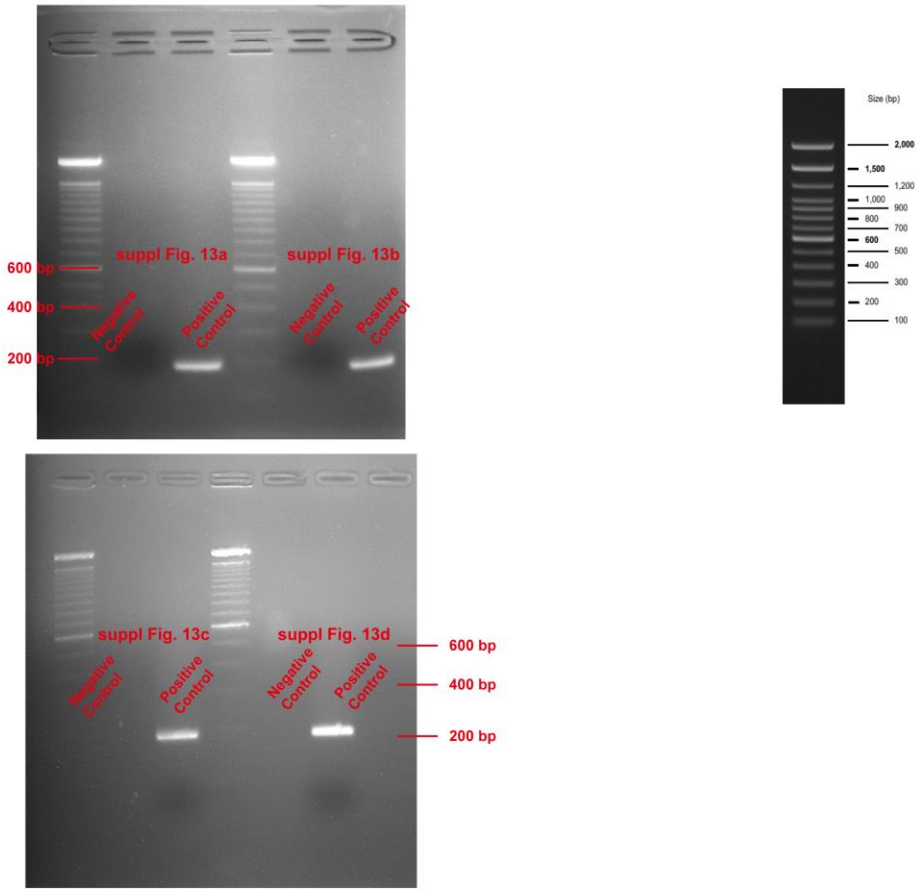

**2% Agrose Gel**

**DNA Marker: Invitrogen 100 bp DNA ladder (Cat. 15628019)**

Supplement: Supplementary file 4 — Source Data [file 41467_2022_33012_MOESM4_ESM.zip › source data/uncropped DNA gels scan/Supplementary Fig. 13a-d uncropped DNA gels.pdf]
